# Reducing pain and discomfort associated with rubber dam clamp placement in children and adolescents: a systematic review and meta-analysis of effectiveness

**DOI:** 10.1186/s12903-023-03115-7

**Published:** 2023-06-16

**Authors:** Elham Afshari, Sedigheh Sabbagh, Fatemeh Khorakian, Alireza Sarraf Shirazi, Alireza Akbarzadeh Baghban

**Affiliations:** 1 Dental Research Center, Faculty of Dentistry, Mashhad University of Medical Sciences, Mashhad, Iran; 2 Dental Materials Research Center and Department of Pediatric Dentistry, Faculty of Dentistry, Mashhad University of Medical Sciences, Mashhad, Iran; 3 Department of Pediatric Dentistry, Faculty of Dentistry, Mashhad University of Medical Sciences, Mashhad, Iran; 4 Proteomics Research Center, Department of Biostatics, School of Allied Medical Sciences, Shahid Beheshti University of Medical Sciences, Tehran, Iran

**Keywords:** Anesthetics, Pain, Rubber dams, Children

## Abstract

**Background:**

The application of rubber dams is a widely accepted method of tooth isolation in dental practice. Placement of the rubber dam clamp might be associated with levels of pain and discomfort, especially in younger patients. The purpose of the present systematic review is to evaluate the efficacy of the methods for reducing pain and discomfort associated with rubber dam clamp placement in children and adolescents.

**Materials and methods:**

English-language literature from inception until September 6^th^, 2022 was searched in MEDLINE (via PubMed), SCOPUS, Web of Science, Cochrane, EMBASE, and ProQuest Dissertations & Theses Database Global for articles. Randomized controlled trials (RCTs) comparing methods of reducing the pain and/or discomfort associated with rubber dam clamp placement in children and adolescents were retrieved. Risk of bias assessment was performed using a Cochrane risk of bias-2 (RoB-2) risk assessment tool and the certainty of evidence was assessed using the Grading of Recommendations Assessment, Development and Evaluation (GRADE) evidence profile.

Studies were summarized and pooled estimates of pain intensity scores and incidence of pain were calculated. The meta-analysis was conducted in the following groups according to type of interventions (LA, audiovisual (AV) distraction, behavior management (BM), electronic dental anesthesia (EDA), mandibular infiltration, inferior alveolar nerve block (IANB), TA), outcome (intensity or incidence of pain), and assessment tool (face – legs – activity – cry – consolability (FLACC), color scale, sounds – motor – ocular changes, and faces pain scale (FPS)):

(a) pain intensity using (LA + AV) vs (LA + BM), (b) pain intensity using EDA vs LA (c) presence or absence of pain using EDA vs LA (d) presence or absence of pain using mandibular infiltration vs IANB (e) Comparing pain intensity using TA vs placebo (f) Presence or absence of pain using TA vs placebo.

Meta-analysis was conducted using StataMP software, version 17.0 (StataCorp, College Station, Texas). Restricted maximum-likelihood random effect model (REML), Mean difference (MD) with 95% confidence interval, and log odds ratio (OR) with 95% CI were calculated were calculated.

**Results:**

Initially, 1452 articles were retrieved. Sixteen RCTs were finally included for reviewing and summarizing. Nine articles with a total of 867 patients were included for quantitative meta-analysis. The differences in pain intensity scores were not significant in any comparison groups (group a: [MD = -0.04 (95% CI =  − 0.56, 0.47), *P* = 0.87, I^2^ = 0.00%], group b: [MD = 0.25 (95% CI = -0.08, 0.58), *P* = 0.14, I^2^ = 0.00%], group c [MD = -0.48 (95% CI = -1.41, 0.45), *P* = 0.31, I 2 = 0.00%], group d: [MD = -0.67 (95% CI = -3.17, 1.83), *P* = 0.60, I 2 = 0.00%], group e: [MD = -0.46 (95% CI = -l.08, 0.15), *P* = 0.14, I 2 = 90.67%], and group f: [MD = 0.61 (95% CI = -0.01, 1.23), *P* = 0.06, I 2 = 41.20%]. Eight studies were judged as having some concern for risk of bias and the remaining studies were considered as low risk for bias. The certainty of evidence was considered medium for all comparison groups.

**Discussion:**

In the present meta-analysis, a considerable difference was obtained between the included studies regarding intervention methods and pain assessment tools and the analysis was performed in groups with small numbers of the studies. Owing to the mentioned variabilities and the small number of studies, the results of the analysis should be interpreted with caution. The indistinguishability of the manifestations of pain/discomfort from fear/anxiety, particularly in children, should also be considered while using the results of the present study.

Within the limitations of the current study, no significant differences were found between the proposed methods for reducing pain and discomfort associated with rubber dam clamp placement in children and adolescents. A larger number of more homogenous studies regarding intervention methods and pain assessment tools need to be conducted in order to draw stronger conclusions.

**Trial registration:**

This study was registered in PROSPERO (ID number: CRD42021274835) and research deputy of Mashhad University of Medical Sciences with ID number 4000838 (https://research.mums.ac.ir/).

**Supplementary Information:**

The online version contains supplementary material available at 10.1186/s12903-023-03115-7.

## Introduction

Operatory field isolation in dentistry is defined as when the operatory field is shielded from oral fluids such as blood, saliva and gingival crevicular fluids and soft tissues such as lips, gingiva, and tongue. There are some goals to achieve with the isolation, leading to the improvement of both patient’s and operator’s comfort and safety [1–3]. Most children do not cooperate well with the process of dental treatment, so establishing and maintaining the isolation is far more challenging for pediatric patients [2]. Different methods have been introduced and explored for operatory field isolation [4–6]. Most commonly used forms of isolation in pediatric patients include: use of fluid absorbents (such as cotton rolls, absorbent papers, gauze or throat shields), rubber dam, saliva ejector, and administration of drugs [2].

Rubber dam is a widely used efficient method of tooth isolation [6–8] and has particularly received most interest during the Covid-19 pandemic, reducing the risk of infection [9, 10]. The technique that is commonly used for rubber dam placement is using stainless steel clamps. The clamp ideally comes into contact with the cervical area of the chosen tooth at four points, usually resulting in gingival retraction [6, 11]. It should be noted that, although there is not too much pressure applied to the gingival tissue, the process can still cause discomfort particularly in children [12]. Studies have declared that around 64 to 80% of children complain about pain and discomfort associated with rubber dam clamp placement which can cause higher levels of dental anxiety, negatively affect the acceptance of the treatment, and cause challenges to the dentist regarding patient’s behavior control [13].

Preventing pain caused by placement of rubber dam clamps is less of an issue when performing non-conservative treatments (e.g., pulp therapy, extensive restorations) that are typically accompanied by local anesthetics. But, in preventative and conservative treatments (e.g., minimally invasive restorations, sealant placement) that are typically performed without using local anesthesia, it is of high importance to establish the operation field isolation with less discomfort. Therefore, researchers have proposed and evaluated various methods to decrease the amount of pain and discomfort associated with clamp placement [14]. At the time of this review, there are no studies systematically summarizing this information, therefore, the objective of the current study is to evaluate the efficacy of the methods for reducing pain and discomfort associated with rubber dam clamp placement in children and adolescents.

## Methods and materials

This article was completed according to the Preferred Reporting Items for Systematic Reviews and Meta-Analyses (PRISMA) guidelines [15]. The study protocol was registered in PROSPERO with the ID number CRD42021274835.

### Review question

The PICO (population, intervention, comparison, outcome) approach was used to formulate the clinical question as follows: “What is the effectiveness of interventions used alone or in combination with local anesthesia to reduce pain and discomfort related to rubber dam clamp placement in children and adolescents (under 18)?”, identifying:

- P _ Children and adolescents (under 18).
- I _ Different interventions used alone or in combination with local anesthesia.
- C _ Among different interventions.
- O _ Reducing pain and discomfort related to rubber dam clamp.

### Eligibility criteria

Eligible studies had to be randomized clinical trials (RCTs) related to procedures that needed rubber dam placement that were published in English. Studies with sufficient homogenous data for meta-analysis were grouped according to their assessment scales and interventions.

Inclusion criteria were as follows:

- At least two pharmacological/non-pharmacological methods (alone or in combination with local anesthesia) have been compared to reduce the pain and/or discomfort associated with rubber dam clamp placement.
- The study’s population were children and/or adolescents up to 18 years old.
- Pain and/or discomfort related to clamp placement has been assessed.

Exclusion criteria were as follows:

- If the evaluated method was applied with any of the following three situations: prescription of analgesic drugs (narcotic/non-narcotic), under sedation or general anesthesia.
- If other innovative methods of isolation rather than conventional latex rubber dam and clamp were assessed.
- Non-randomized and quasi-randomized studies.

### Search strategy, data collection, and risk of bias assessment

The electronic search was conducted through the six following databases: MEDLINE (via PubMed), SCOPUS, Web of Science, Cochrane, EMBASE, and ProQuest Dissertations & Theses Database Global until September 6^th^, 2022 by one experienced researcher (A.S) (Table 1). Reference articles were managed by using EndNote 20 (Clarivate, Philadelphia, PA, USA). A manual search was performed for the reference lists of all eligible studies for additional eligible studies.

**Table 1:** Databases and search strings

Database	Search string
MEDLINE (via PubMed)	“(Clamp OR "Rubber Dams"[Mesh] OR (rubber dam*)) AND (gingiva* OR gum OR "Gingiva"[Mesh] OR dent* OR teeth OR tooth OR "Tooth"[Mesh]) AND (pain OR "Pain"[Mesh] OR discomfort)”
Other databases (EMBASE, Web of Science (All databases), SCOPUS, Cochrane Central register of Controlled Trials (CENTRAL), Cochrane Database of Systematic Reviews, and ProQuest Dissertations & Theses Database Global)	(Clamp OR (rubber dam*)) AND (gingiva* OR gum OR dent* OR teeth OR tooth) AND (pain OR discomfort

Two independent reviewers (E.A and F.K) screened the title and abstract of each record, and extracted the following data for each eligible study: first author, year of publication, country of origin, trial design, patients’ age and gender, intended dental treatment, initial child behavior, previous dental history, intervention, comparison, tooth type and eruption status, assessment scales, and primary results. Any disagreement between the two reviewers, was resolved by the supervisor (S.S). Authors were contacted to provide raw data if necessary.

A Cochrane risk of bias-2 (RoB-2) risk assessment tool was applied [16]. All included studies were assessed independently by the two reviewing authors (E.A and F.K) who were not blinded to identifying details of articles. Considering the design of the studies, intention-to-treat (ITT) or per-protocol (PP) analyses were conducted. The RoB-2 scale is organized into five domains: randomization process, deviation from intended intervention, missing outcome data, measurement of outcome, and selection of reported result.

Certainty of evidence for each group was assessed using the Grading of Recommendations Assessment, Development and Evaluation (GRADE) evidence profile [17] and was classified as “very low”, “low”, “moderate”, and “high” based on identified risk of bias, inconsistency, indirectness, imprecision, and publication bias.

### Data analysis

Meta-analysis was conducted using StataMP software, version 17.0 (StataCorp, College Station, Texas) and was performed according to the restricted maximum-likelihood random effect model (REML) irrespective of heterogeneity status of included studies. Both intensity and incidence of pain were considered as the outcomes of this review. Mean difference (MD) and its corresponding 95% confidence interval (95% CI) were calculated for the former outcome. For a study to be eligible for the meta-analysis of this outcome, the mean and standard deviation for pain intensity as well as sample size must be available. For the latter outcome, log odds ratio (OR) and its corresponding 95% CI were calculated. Again for a study to be eligible for the meta-analysis, the number of subjects with and without pain experience during clamp placement must be available. Statistical analyses were only performed for those groups of studies with similar assessment scales and interventions. Heterogeneity between the estimates was evaluated by Cochrane’s test (I^2^ test) and I^2^ > 50% was considered as high heterogeneity.

## Results

### Study selection and data retrieval

**Fig. 1:**
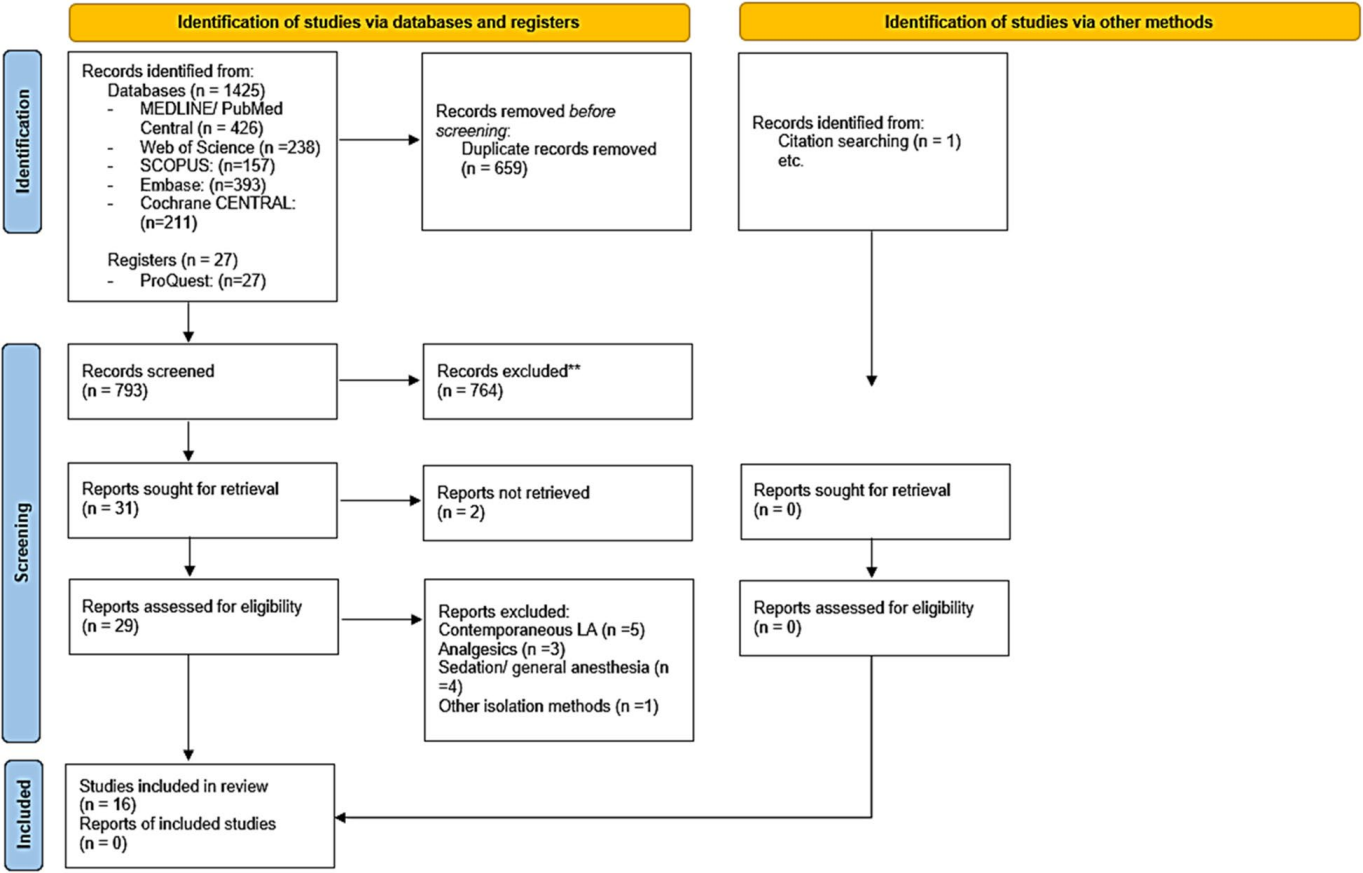
Flow diagram of the study process according to the Preferred Reporting Items for Systematic Reviews and Meta-analyses (PRISMA) statement

Of the 1452 articles identified in the initial search, 16 studies fulfilled the inclusion criteria and were eligible for analyses [12, 13, 18–31], of which nine reported sufficient data for meta-analysis in six different groups (Fig. 1) [13, 20, 22, 26–28, 31].

Overall, 867 children and adolescents aged 4–17.4 years were included in the analysis. The included studies were published between 1989 [30] and 2022 [19]. Nine of the included articles described split-mouth studies [12, 13, 18, 24, 26–29, 31], three reported crossover studies [19, 20, 22], one combined both split-mouth and crossover designs [30], and three were parallel studies [21, 23, 25].

As regards the type of anesthesia, local anesthesia (LA) was used in 10 of the included studies (Table 2) and topical anesthesia (TA) was used in six studies (Table 3). Variable dental treatments were reported in the selected studies, with rubber dam application as a part of them, such as sealant placement, restorative treatments, pulpotomy, and pulpectomy.

**Table 2:** Characteristics of the included studies in which local anesthesia was used

Authors, year of publication, country	Trial design	Age (years), gender (%)	Initial child behaviour/ Previous dental experiences	Intended dental treatment(s)	Interventions for pain/discomfort reduction		Tooth type /eruption status	Assessment tool(s) for Pain/discomfort	Effectiveness of interventions during rubber dam application/clamp placement
					**Category** **(#n)**	**Descriptions**			
**Zaidman et al., 2022, **[19]** Israel**	Crossover	4–12 (8.34 ± 2.1) M:72%- F:28%	Fully cooperative (Frankl grade 4) during examination /Y: 93%, N: 7%	Routine pediatric dental treatment that included IANB and rubber dam placement	LA + VR distraction (*n* = 29 C)	VR goggles: Oculus Go VR goggles from Facebook Technologies (Oculus Go virtual reality goggles, Meta Quest, Facebook Technologies, LLC) Content: 2 cartoon series, one children’s show (average screening time: ~ 30 min)	NM/NM	1-Wong-Baker FACES Pain Rating Scale (self-report) (0–10) 2- MBPS (facial expression, crying, and movement) (0–9)	The use of VR glasses resulted in lower mean scores of the Wong-Baker FACES Pain Rating Scale (*P* = 0.005) and MBPS parameters (Face (*P* = 0.005), Cry (*P* = 0.029), and Movement (*P* = 0.028)). The order of using VR goggles had no significant effects on pain levels (*P* > 0.05)
					LA (*n* = 29 C)	Treatment was performed without the VR goggles			
**﻿Garrocho-Rangel et al., 2018, **[20]** Mexico**	Crossover	5–8 (6.2 ± 1.3) M: %55.6-F: %44.4	﻿Frankl scale: -Abstract: II (%42.8) and III (%57.2)/ -Methods section:I (%42.8) and II (%57.2)/no previous dental experience	Cavity preparation, pulpotomy/SSC	LA + AV distraction (*n* = 36 T/C)	﻿Video Eyeglasses/Earphones System: Virtual Private Theater Video Glasses (Chinavision®; Kowloon, Hong Kong, China), with earphones	﻿Upper or lower primary molars/NM	FLACC scale (0–10)	Pain perception following rubber dam placement was NSS (*P* = 0.7)
					﻿LA + BM (*n* = 36 T/C)	﻿Other (non-aversive) behavioural techniques, such as the “tell-show-do” method and continuous verbal communication			
**Alamoudi et al., 2016, **[21]** Saudi Arabia**	Parallel	5–9 M: 42.9%-F: 57.1%	Positive or definitely positive according to the Frankl behavior classification scale/NM	Pulpotomy	LA-IANB (*n* = 31 C)	Traditional IANB using traditional syringe and lidocaine HCl 2% with epinephrine (1:100,000)	Primary mandibular second molars/NM	SEM scale (3–12)	Difference between three techniques for clamp placement was NSS (*P* = 0.635). Moreover, the correlation between age and anesthesia effectiveness for clamp placement was weak (r = 0.145)
					CCLAD-IANB (*n* = 30 C)	IANB using CCLAD performed with Single Tooth Anesthesia System (manufactured by Milestone Scientific) and lidocaine HCl 2% with epinephrine (1:100,000)			
					CCLAD-ILA (*n* = 30 C)	ILA injection using CCLAD performed with Single Tooth Anesthesia System (manufactured by Milestone Scientific) and lidocaine HCl 2% with epinephrine (1:100,000)			
**Mitrakul et al., 2015, **[22]** Thailand**	Crossover	5–8 (6.9 ± 0.9) M: 38%- F: 62%	Frankl scale 3 or 4 (positive behavior patients)/Y: 62% (with LA: 21%, without LA: 41%), N: 38%	Restorative treatment (amalgam and composite fillings, SSCs, pulpotomy, pulpectomy, and Ext)	LA + AV distraction (*n* = 42 C)	AV eyeglasses composed of headmounted display (video glasses cool vision 3—Shenzhen Longway Vision Technology Co. Ltd, Shenzhen, China) and in-ear headphones	Maxillary or mandibular molars /NM	1- FPS-R (0–10) 2- FLACC scale (0–10)	FLACC scores for the effect of wearing AV eyeglasses in both groups (the sequence of using AV eyeglasses was different) were NSS (*P* = 0.476). This was also true for period effect (*P* = 0.351) and carry-over effect (*P* = 0.806)
					LA + BM (*n* = 42 C)	tell-show-do, positive reinforcement, and conventional distraction ﻿(deep breath or breath counting)			
**Yilmaz et al., 2011, **[25]** Turkey**	Parallel	6–8 ﻿ (7.2 ± 0.6) M: 50%- F: 50%	Frankl ﻿behavior rating scale 3 and 4 (positive and ﻿definitively positive)/ NM	Pulpotomy	LA- Articaine (*n* = 81 C/T)	injection of 1 mL 4% articaine HCl with 1:100,000 epinephrine (Ultracaine DS®, Aventis, Istanbul, Turkey) with a five-min waiting time	Maxillary or mandibular primary molars/NM	FHTLC-pain-related behaviors (Facial expression, eye squeezing, hand movements, torso movements, leg movements, crying)	The differences between the two LA agents were NSS, irrespective of administration technique (P > 0.05)
					LA- Prilocaine (*n* = 81 C/T)	Injection of 1 mL of 3% prilocaine HCl with 1.08 μg felypressin (Citanest® Octapressin, AstraZeneca, Istanbul, Turkey) with a 5-min waiting time			
**Yassen, 2010, **[26]** Iraq**	Split-mouth	6–9, M: 43%- F: 57%	Children whose behaviour interfered with an assessment of discomfort or pain were excluded/ NM	Restoration (Class III, IV, and V), pulpotomy, Ext	LA-infiltration (*n* = 36 C/T)	﻿Needle was advanced in the mucobuccal fold towards the apex of the teeth. 1.2 mL of 2% lidocaine HCl with epinephrine 1:80,000 (Lignospan® Septodont; Mazamet Cedex, France) was used with a 5-min waiting period	Mandibular primary canines/ NM	﻿Assessments were based on ﻿sounds, motor, and ocular changes indicating pain (hand and body tension, eye movement, verbal complaints, tears)	The differences in pain between two anaesthetic techniques during rubber dam placement for restorative treatments (*P* = 0.54) and pulpotomies (*P* = 1) were NSS
					LA-IANB (*n* = 36 C/T)	﻿ ~ 1.6 mL of 2% lidocaine, 1:80,000 epinephrine (Lignospan) was administered			
**Baghdadi, 1999, **[27]** Syria**	Split-mouth	6–12 (﻿10.21 ± 1.4) M:39.3%- F: 61.7%	Cooperative enough to follow the instructor’s directions + ﻿histories of compliance at previous clinic visits/ Y	Class I amalgam restorations	EDA (*n* = 28 C)	(3 M Dental Electronic Anesthesia System 8670, 3 M Dental Products, St Paul, Minn)	Mandibular second primary molars and maxillary and mandibular first permanent molars/NM	Color scale/ SEM scale	The differences between LA and EDA regarding subjective and objective pain were NSS (P > 0.05)
					LA (*n* = 28 C)	Conventional LA using ﻿1.2 mL or 2/3 a cartridge of ﻿2% lidocaine with 1:80,000 Epinephrine (Weimer Pharma GmbH, Rastatt, Germany) with 5-min waiting time			
**Oulis et al., 1996, **[28]** Greece**	Split-mouth	3–9 M: 47%- F: 53%	Cooperative at the initial visit/ Y	Class I and II amalgam restorations, SSC, formocresol pulpotomies, Ext	LA-infiltration (*n* = 152 T)	Mandibular infiltration was administered using ﻿1.7 ml of lidocaine HCl 2% with epinephrine 1:100,000 (Xylestesin Forte, Espe Seefeld/Oberbay, Germany) in the mucobuccal fold between the roots of the first and second primary molars and in the mesial and distal papillae, with a 5-min waiting period	Mandibular primary molars/ NM	Presence or absence of pain based on sounds, and motor and ocular changes indicating pain (hand and body tension, eye movements indicating pain, verbal complaints, tears, and hand and body movements)	The difference between the two techniques was NSS
					LA-IANB (*n* = 152 T)	Mandibular block was administered using the conventional technique and 1.7 ml of the anesthetic solution in addition to long buccal nerve injection			
**teDu et al., 1993, **[18]** USA**	Split-mouth	6–12 NM	Cooperative/ NM	Preventive resin restorations	EDA (*n* = 27 C)	TENS (﻿Spectrum Max-SD®, Medical Designs, Westerville, OH; setting: pulse rate: 110 Hz, a normal mode pulse width of 225 microseconds, ﻿amplitude level: 7–12 mA, waveform: asymmetrical, rectangular, biphasic pulse with a net zero D.C. component). Disposable electrode pads (Dentrode 37®, The Electrode Store, Yucca Valley, CA) were used with a 5-min waiting time	Primary (22.2%)/permanent (77.8%) molars/NM	Eland Color Scale	﻿Differences in pain perception between EDA and LA ﻿regarding effectiveness in controlling pain perception were NSS (P > 0.05)
					LA (*n* = 27 C)	Traditional/conventional LA (maxilla: infiltration + lingual soft tissue anesthesia; mandible: ﻿Long buccal, inferior alveolar and lingual nerve blocks) using 2% lidocaine with 1:100,000 epinephrine (Xylocaine®, Astra, Westborough, MA) with 5 min waiting time			
**Abdulhameed et al., 1989, **[30]** USA**	Crossover/split-mouth	8–14 (11 ± 16) M: 50%- F: 50%	NM/ NM	Sealant placement	EDA-9 kHz (*n* = NM)	﻿Peripheral electrical stimulation, 9/12/15/20/25 kHz electrical stimulus (Electro-Dental Anesthesia, Hauser Laboratories Inc., Boulder, Colo.), setting: waveform: trains of symmetrical rectangular wave pulses, maximum amplitude: 30 mA), 3 min waiting time	Maxillary and mandibular first (80%) or second (20%) permanent molars/NM	VAS (self-report and objective), heart rate using pulse oximeter	The increase in the heart rate was significantly less during electrical stimulation compared with sham stimulation (*P* < 0.05). The differences in subjects’ or investigator’s VAS scores between electrical and sham stimulation as well as the effectiveness of electrical stimulation between the 5 frequencies were NSS
					EDA-12 kHz (*n* = NM)				
					EDA-15 kHz (*n* = NM)				
					EDA-20 kHz (*n* = NM)				
					EDA-25 kHz (*n* = NM)				
					Placebo (*n* = NM)	The device was not functional			

*IANB* Inferior Alveolar Nerve Block, *NM* Not mentioned, *min* Minute(s), *NSS* Not statistically significant, *F* Female, *M* Male, *AV* Audiovisual, *FLACC* Face, Legs, Activity, Cry, Consolability, *CCLAD* Computer-controlled local anesthetic delivery system, *SEM* sounds, eyes, and motor, *ILA* Intraligamental anesthesia, *FPS-R* Faces Pain Scale-Revised, *SSC* Metallic preformed crowns/stainless steel crown, *Ext* Extractions, *VAS* Visual analog scale, *TENS* Transcutaneous electrical nerve stimulator, *EDA* Electronic dental anesthesia, *T* Teeth, *C* Children, *BM* Traditional behaviour management, *Y* Yes, *N* No, *MBPS* Modified Behavioral Pain Scale, *VR* Virtual reality

**Table 3:** Characteristics of the included studies in which topical anesthesia was used

Authors, year of publication, country	Trial design	Age (years), gender (%)	Initial child behaviour/ Previous dental experiences	Interventions for reduction of pain/discomfort		Tooth type /eruption status	Assessment tool(s) for Pain/discomfort	Effectiveness of interventions during rubber dam application/clamp placement
				**Category** **(#n)**	**Descriptions**			
﻿Wambier et al., 2018, [32] Brazil	Split-mouth	8–12 (﻿10.8 ± 0.5), M: 48%- F: 52%	NM/NM	TA gel-Exp (*n* = 81 T/C)	Liposomal ﻿ thermo-sensitive Anesthetic Gel ﻿composed of 5% lidocaine and prilocaine/applied ~ 2 mm beyond the gingival marginal for 2 min	﻿Mandibular 6 s/fully erupted	Odds of having pain: positive/negative Pain intensity 1- Wong-Baker FACES scale (0–5) 2- a 11-point numerical scale	Pain experience was NSS (OR: 0.7, %95 CI = 0.3–1.8). ﻿Pain intensity was statistically different for both assessment scales (*P* = 0.023 for numerical scale, *P* = 0.013 for Wong-Baker FACES scale)
				Placebo (*n* = 81 T/C)	Placebo gel/ applied ~ 2 mm beyond the gingival marginal for 2 min			
Wambier et al., 2018, [32] Brazil	Split-mouth	8–12 (10.4 ± 1.0), M: 52%-F: 48%	NM/ NM	TA gel-Exp (*n* = 82 T/C)	A light-cured anesthetic gel (Patent-BR 1020160077249) containing tetracaine hydrochloride (5%), inhibitor, monomers, photoinitiator, co-initiator, dye and inert load. The gel was applied ~ 2 mm beyond the gingival margin, left untouched for 15 s before light curing with an LED device (Radii-cal, 1,200 mW/cm^2^). The waiting time was 30 s	Mandibular 6 s/ fully erupted	Absolute risk of pain: yes/no Pain intensity: 1- Facial expression Wong-Baker scale (0–5) 2-FLACC scale (0–10) 3- 11-point numeric rating scale (0–10)	The experimental light-cured anesthetic gel reduced the risk and intensity of pain compared to the placebo gel (*p* < 0.001)
				Placebo (*n* = 82 T/C)	Manipulated similar to the experimental TA gel			
**Coudert et al., 2014, **[23]** France**	Parallel	6–15/NM	Uncooperative children were excluded/NM	TA-com (*n* = 18)	2% lidocaine HCl (20 mg/1 g cream) cream, rubbed for 1 min and wait for 3 min, excipients: preservative (benzalkonium chloride), flavoring (thymol, aromatic oils), and emollients (liquid paraffin) with no anesthetic or analgesic properties	NM/NM	A 100-mm VAS	﻿Pain reduction was significantly greater in the TA group than in the placebo group (*P* < 0.005)
				Placebo (*n* = 21)	Placebo cream (with an identical excipient composition—20 mg water was used in place of the lidocaine HCl), rubbed for 1 min and wait for 3 min			
**Yoon & ﻿Chussid, 2009 **[24]**, USA**	Split-mouth (pilot study)	7–12, M: 40%- F: 60%	Cooperative patients ﻿whose behavior was not a contraindication to sealant placement (without behavior problems)/NM	TA-Com (*n* = 45 C)	Oraqix gel ﻿(2.5% lidocaine, 2.5% prilocaine, Dentsply Pharmaceutical, York, Pa), applied around the entire gingival sulcus, ~ 1/4 carpule or 0.4 g was used (depressing the applicator paddle 5 times) with 2 min waiting time	6 s/ NM	Modified FPS	The overall difference between the two TA agents in mean FPS ratings was NSS (*P* = 0.27). Oraqix was more effective in 9-to-12- year-old children (*P* = 0.04)
				TA-Com (*n* = 45 C)	20% benzocaine gel ﻿(Patterson Dental, Saint Paul, Minn), applied ﻿on the gingiva surrounding the entire tooth with a Q-tip applicator with 2 min waiting time			
**Lim & ﻿Julliard, 2004, **[12]** USA**	Split-mouth	6–12 M: 42%- F: 58%	Children with behavioral difficulties on prior dental visits were excluded/ most were cooperative/NM	TA-Com (*n* = 31)	0.5 g of EMLA (2.5% lidocaine and 2.5% prilocaine) cream (﻿Astra Pharmaceuticals)/ applied on attached gingiva for 5 min	Maxillary (65%) and mandibular (35%) 6 s /completely or partially erupted	FPS	The mean FPS score for EMLA was significantly lower than that for non-EMLA (*P* < .001)
				Placebo (*n* = 31)	0.5 g of Vaseline/applied for 5 min on gingiva			
**Stecker et al., USA, **[29]** 2002**	Split-mouth	6.4–17.4 (11.3 ± 3.5) M: 58%- F: 42%	Cooperative (based on dental progress notes)/ NM	TA-Com (*n* = 37 T/28 C)	Mucosal adhesive patch (20% lidocaine) ﻿contained 46.1 mg of lidocaine in a bioadhesive matrix (DentiPatch), half of the patch was applied ~ 1 mm below the facial/lingual gingiva for 5 min	Maxillary or mandibular first and second premolars and permanent molars/ NM	VAS (0–100)	The differences in VAS/pain scores were NSS (*P* > .18)
				TA-Com (*n* = 37 T/28 C)	Hurricaine Dry Handle Swab ﻿contained 0.25 mg of 20% benzocaine, ﻿1 swab was applied to both the facial and lingual gingiva in equal amounts by alternating for 1 min			

*6 s* First permanent molars, *TA* Topical anesthetic, *NM* Not mentioned, *min* Minute(s), *OR* Odds ratio, *NSS* Not statistically significant, *F* Female, *M* Male, *FLACC* Face, Legs, Activity, Cry, Consolability, *FPS* Faces Pain Scale, *VAS* Visual analog scale, *EMLA* Eutectic mixture of local anesthetics, *C* Children, *T* Teeth, *Exp* Experimental, *Com* Commercial

Included RCTs were performed on different types of teeth. Five studies were performed on permanent molars [12, 13, 24, 30, 31], four studies were performed on primary molars [20, 21, 25, 28], two studies included both primary and permanent molars [18, 27], one study was performed on primary canines [26], one study was performed on permanent molars and premolars [29]. Tooth type was not exactly mentioned in 3 studies [19, 22, 23].

### Risk of bias assessment

**Fig. 2:**
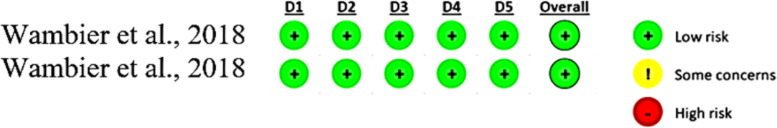
RoB-2 Summary for included studies (PP). ( +) denotes low risk or bias; (-) denotes high risk of bias; and (!) denotes moderate risk of bias. PP: intention to treat

**Fig. 3:**
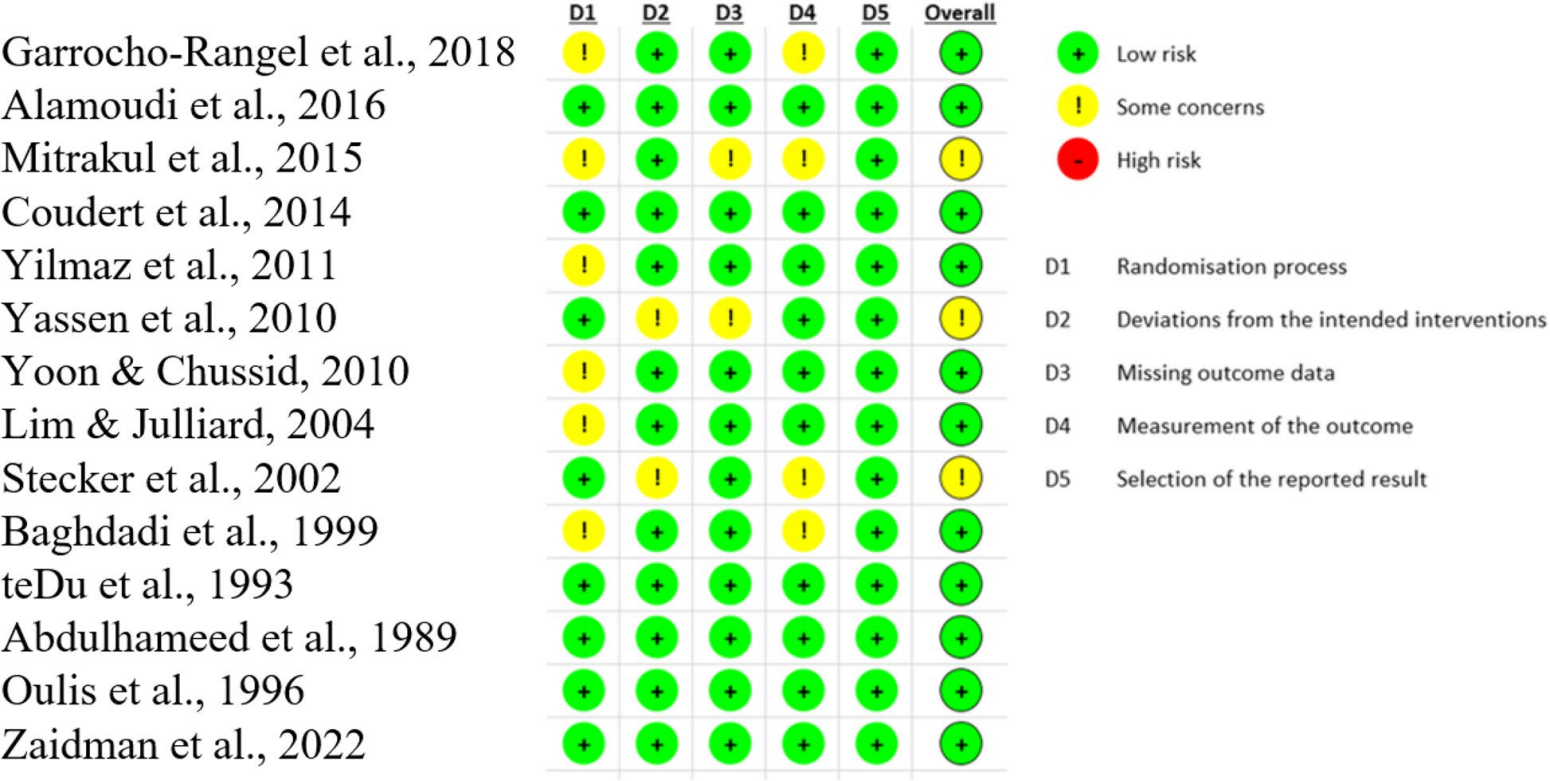
RoB-2 Summary for included studies (PP). ( +) denotes low risk or bias; (-) denotes high risk of bias; and (!) denotes moderate risk of bias. PP: per-protocol

The results of the risk of bias assessment of the included studies are summarized in Fig. 2 (ITT) and Fig. 3 (PP). Three studies were judged as having some concern for risk of bias, [22, 26, 29] and the remaining studies were considered as low risk for bias. The reasons leading to some concern for risk of bias in the three mentioned studies are as follows: Two studies had some concern in terms of randomization process [22, 26] in which data was not available for all, or nearly all, participants randomized. In two studies, there was no information about whether the allocation sequence was concealed until the interventions were implemented [22, 29]. Two studies were considered to have deviations from intended intervention as the operators, participants, or outcome assessors [26, 29] were not or could not be blinded to the interventions which could potentially influence assessment of the outcome. One study even reported elimination of seven participants from the study due to their because behavior which prevented reasonable pain evaluation [26]. As children demonstrating more negative behavior might have experienced more pain and discomfort, the missing outcome data can bias the result. Also in one study [26], previous dental history was not mentioned which can be a potentially confounding factor.

Some concern for bias was also observed in studies considered as low risk of bias, however there were not considerable in terms of importance or in number. In one study [29], there was some concern regarding the baseline differences between intervention groups as it was stated in the article that the baseline Frankle ratings [33] had little variations and therefore no statistical analysis was performed.

### Certainty of evidence

According to the GRADE approach, the certainty of evidence was considered as “medium” in all meta-analysis groups. Regarding that only RCTs were included in this study, the initial certainty of all groups were rated as high and was downgraded in the suspicion of risk of bias, inconsistency, indirectness, imprecision, and publication bias. The main reason for downgrading the evidence was due to imprecision (small sample size and wide confidence intervals).

### Qualitative analysis and quantitative synthesis of the results

Nine of the selected studies reported sufficient homogenous data for meta-analysis [13, 20, 22, 26–28, 31]. The RCTs were grouped into six categories according to type of interventions (LA, audiovisual (AV) distraction, behavior management (BM), electronic dental anesthesia (EDA), mandibular infiltration, inferior alveolar nerve block (IANB), TA), outcome (intensity or incidence of pain), and assessment tool (face – legs – activity – cry – consolability (FLACC), color scale, sounds – motor – ocular changes, and faces pain scale (FPS)):

- Comparing pain intensity using (LA + AV) vs (LA + BM) – FLACC scale

**Fig. 4:**
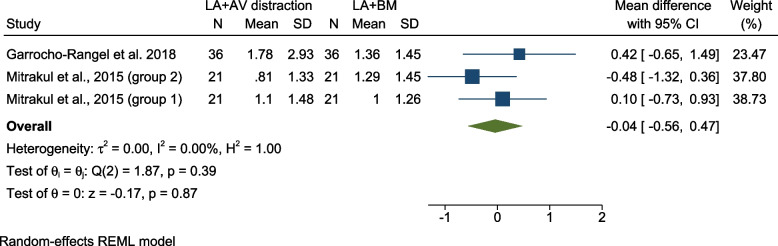
Forest plot Comparing pain intensity using (LA + AV) vs (LA + BM) – FLACC scale; LA: Local Anesthesia, AV: Audiovisual, BM: Behavior Management, N: Number, SD: Standard Deviation, CI: Confidence Interval, REML: restricted maximum-likelihood

Two studies compared pain intensity while using LA associated with AV distraction, vs LA associated with BM [20, 22]. One study was judged to be at low risk for bias [20] and the other one was judged to have “some concern” for bias [22]. Both studies were crossover RCTs conducted on 5–8-year-olds. Results were separately reported for two independent groups in one study according to the sequence of interventions, [22], thus the data was analyzed in three groups/datasets (Fig. 4). The pooled results showed no significant difference of pain intensity regarding clamp placement [MD = -0.04 (95% CI =  − 0.56, 0.47), P = 0.87, I^2^ = 0.00%].

(b): Comparing pain intensity using EDA vs LA – color scale.

**Fig. 5:**
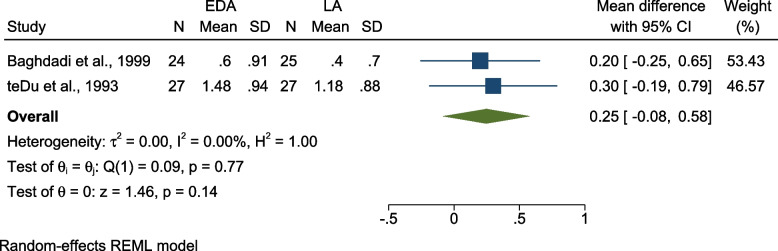
Forest plot comparing pain intensity using EDA vs LA – color scale; EDA: Electronic Dental Anesthesia, LA: Local Anesthesia, N: Number, SD: Standard Deviation, CI: Confidence Interval, REML: restricted maximum-likelihood

Two studies compared pain intensity using EDA and LA [18, 27]. Both studies were split-mouth RCTs conducted on 6–12-year-olds and were judged to be at low risk for bias. The pooled results showed no significant difference of pain intensity regarding clamp placement [MD = 0.25 (95% CI = -0.08, 0.58), P = 0.14, I^2^ = 0.00%] (Fig. 5).

(c): Comparing pain incidence using EDA vs LA – color scale

**Fig. 6:**
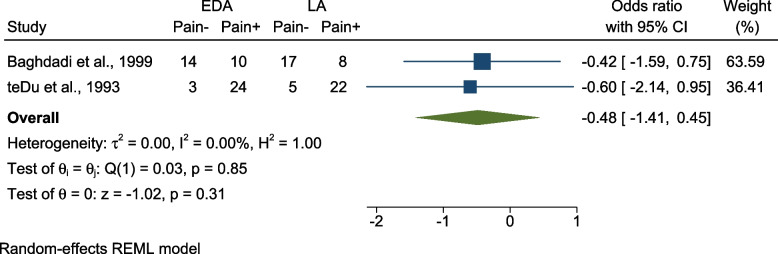
Forest plot Comparing presence or absence of pain using EDA vs LA – color scale; EDA: Electronic Dental Anesthesia, LA: Local Anesthesia, N: Number, SD: Standard Deviation, CI: Confidence Interval, REML: restricted maximum-likelihood

Incidence of pain while using EDA vs LA was assessed in the same studies mentioned in part (b) [18, 27]. The pooled results showed no significant difference in pain incidence regarding clamp placement [OR = -0.48 (95% CI = -1.41, 0.45), P = 0.31, I 2 = 0.00%] (Fig. 6).

(d): Comparing pain incidence using mandibular infiltration vs IANB – sounds, motor, and ocular changes

**Fig. 7:**
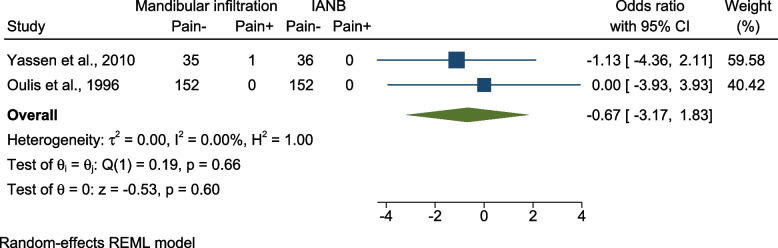
Forest plot Comparing presence or absence of pain using mandibular infiltration vs IANB – sounds, motor, and ocular changes; IANB: Inferior Alveolar Nerve Block, CI: Confidence Interval, REML: restricted maximum-likelihood

Two studies compared the incidence of pain while using mandibular infiltration vs IANB [26, 28]. Both studies were split-mouth RCTs. One was conducted on 3–9-year-olds and was judged to have “some concern” for bias [26] and the other one was conducted on 6–9-year-olds with low risk for bias [28]. The pooled results revealed no significant difference in pain incidence regarding clamp placement [OR = -0.67 (95% CI = -3.17, 1.83), P = 0.60, I 2 = 0.00%] (Fig. 7).

(e): Comparing pain intensity using TA vs placebo – FPS

**Fig. 8:**
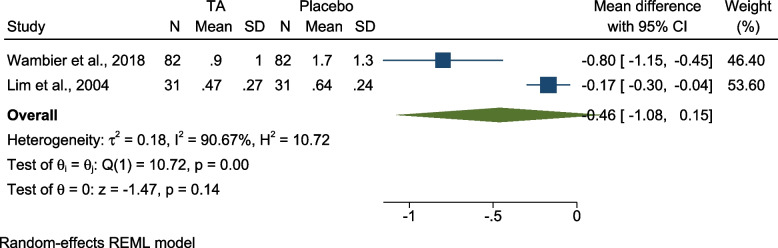
Forest plot Comparing pain intensity using TA vs placebo – FPS; TA: Topical Anesthesia, N: Number, SD: Standard Deviation, CI: Confidence Interval, REML: restricted maximum-likelihood

Two studies compared pain intensity while using TA vs placebo [12, 31]. Both studies were split-mouth RCTs judged as low risk of bias. One was conducted on 8–12-year-olds [31] and the other on 6–12-year-olds [12]. The pooled results revealed no significant difference of pain intensity regarding clamp placement [MD = -0.46 (95% CI = -l.08, 0.15), P = 0.14, I 2 = 90.67%] (Fig. 8).

(f): Comparing pain incidence using TA vs placebo – FPS

**Fig. 9:**
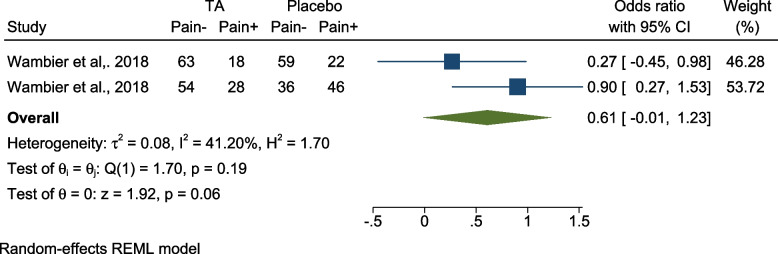
Forest plot Presence or absence of pain using TA vs placebo – FPS; TA: Topical Anesthesia, CI: Confidence Interval, REML: restricted maximum-likelihood

Two studies compared the incidence of pain while using TA vs placebo [13, 31]. Both studies were split-mouth RCTs conducted on 8–12-year-olds and judged as low risk for bias. The pooled results showed no significant difference of pain incidence regarding clamp placement [OR = 0.61 (95% CI = -0.01, 1.23), P = 0.06, I 2 = 41.20%] (Fig. 9).

#### Local anesthesia

Researchers in one of the reviewed studies used virtual reality (VR) goggles as a distraction method for reducing pain and discomfort during routine dental treatments; and the results were assessed using Wong-Baker FACES Pain Rating Scale and MBPS (facial expression, crying, and movement) parameters. They reported significantly lower pain intensity of clamp placement while using VR goggles [17]. Researchers in one of the selected studies compared three methods of IANB using a computer-controlled local anesthesia delivery system (CCLAD), traditional IANB, and infiltration local anesthesia using CCLAD. They used a sound, eye, motor (SEM) scale as a pain assessment tool and reported no significant difference [21]. Investigators in another study compared the effect of two different LA solutions (i.e. Articaine vs Prilicaine) [25]. They also reported no significant difference during clamp placement. One study compared the effect of different frequencies of peripheral electrical stimulation (PES) on pain reduction during sealant placement and reported a significantly lower increase in the heart rate using PES compared with sham stimulation [30]. However, the differences in subjects’ or investigator’s visual analogue scale (VAS) scores between electrical and sham stimulation as well as the effectiveness of electrical stimulation between the five frequencies were not significant.

#### Topical anesthesia

One of the included studies compared the effect of lidocaine cream with placebo cream, using VAS. They reported a significantly greater reduction of pain while using lidocaine cream [23]. Investigators in one of the selected studies compared Oraqix (2.5% lidocaine, 2.5% prilocaine) gel with benzocaine gel, using FPS. They reported no significant overall difference between the two groups. However, Oraqix showed significantly less pain in 9-to-12- year-old children [24]. Another included study compared the effect of lidocaine mucosal adhesive patch with Hurricaine Dry Handle Swab ﻿(0.25 mg of 20% benzocaine). VAS revealed no significant difference of pain reduction regarding clamp placement [29].

## Discussion

To the best of our knowledge, this is the first systematic review and meta-analysis to assess the efficacy of interventions for reducing pain and discomfort related to rubber dam clamp placement in children. In the current study, data from 16 RCTs were assessed. The meta-analysis demonstrated that there were no significant differences between proposed interventions ((LA + AV), (LA + BM), EDA, LA, mandibular infiltration, IANB, TA, and placebo)) regarding incidence and intensity of pain associated with clamp placement in children. The current review targeted pain regarding rubber dam clamp placement which can be utilized as part of different dental procedures. The selected studies included conservative procedures such as preventive restorations and sealant placement without LA, to more invasive procedures such as pulpotomy or pulpectomy of vital inflamed dental pulp where LA is inevitable. The perceived pain associated with clamp placement is certainly affected by the use of LA.

The current study aimed to assess the efficacy of interventions for reducing pain and discomfort. However, dental fear, dental anxiety, and pain are strongly related concepts and their manifestations should be distinguished, especially in children [20]. Some of the included studies reported physiological changes such as increasing heart rate as indications of anxiety besides subjective and/or objective indices of pain [25]. Thus, variables regarded as anxiety indices in the articles were not included in the study. Pain and discomfort are also two concepts that cannot be easily distinguished and some literature use the words interchangeably [30]. Therefore, both pain and discomfort were used for keyword search over databases and studies reporting discomfort associated with dental procedures were also included in the review [17].

Among several methods developed for tooth isolation, rubber dam, cotton rolls, and Isolite are most studied [34]. Recent studies have shown little or no advantage in success rate of restorative treatments using rubber dam rather than cotton rolls or Isolite [34, 35]. However, rubber dam is a widely used method of tooth isolation which is considered by many as the standard of care or the most efficient method of tooth isolation [6–8] and has particularly received interest during the Covid-19 pandemic, reducing the risk of infection [9, 10]. A rubber dam is usually held in place by using clamps. Several types of rubber dam clamps have been also developed and marketed, and the most commonly used are stainless steel clamps [11]. Thus, in order to include more homogenous studies, articles introducing methods of tooth isolation other than the common rubber dam and stainless steel clamps were excluded from the review [36].

The literature has declared that previous dental experience is highly related to the pain perception of child patients [37]. Presence or absence of previous dental experience was noticed in some of the included studies [19, 20, 22, 27, 28], while others did not mention this factor as inclusion/exclusion criteria. Versloot et al. assessed the effect of age, injection site, child’s dental history, dental anxiety and local anesthetic receptor site on pain perception in children. They reported that children’s level of dental anxiety was the most influencing factor on pain level for younger children and having previous dental experience was the most influencing factor in older children [37].

A child’s uncooperativeness clearly interferes with assessing the manifestations of dental pain for the dentist and makes unreliable the results of self-reported pain scales such as VAS and color scale. The cooperativeness of included patients was noted by the researchers in the selected studies. The included studies mentioned the patients as being cooperative or scored 3 or 4 on the Frankl scale [33] except two articles, one of which did not mention the level of cooperativeness [30] and the other one reported different Frankl scale ratings in the abstract and the methods Sect.  [20]. Status of tooth eruption is determinative in discomfort associated with clamp placement. However, the eruption status was not mentioned in most of the selected studies. Special types of clamps are developed for partially erupted teeth, however, the clamps are used for subgingival placement which can cause pain [38, 39].

A noteworthy feature of the studies included in this review was the considerable difference among the studies regarding intervention methods and pain assessment tools, leading to significant heterogeneity which may affect the final outcome of the study. Also, some concern was identified for risk of bias for eight of the included studies and a medium certainty of evidence identified was identified in all comparison groups, which may affect the confidence of the results. Another limitation of the current review is the small number of the studies in each group. Owing to the mentioned variabilities and the small number of studies, the results of the analysis should be interpreted with caution. The indistinguishability of the manifestations of pain/discomfort from fear/anxiety, particularly in children, should also be considered while using the results of the present study.

Besides the mentioned limitations, the current systematic review has several strengths. The main strength of this study is that it is the first systematic review on this topic. The study included only RCTs, thus it can be regarded as high-level evidence. Having a focused clinical research question, conducting a comprehensive literature search in a number of large and reliable databases, and using RoB-2 which is a validated and reliable risk assessment tool are other strengths of this study.

Owing to the small number of studies in each analytical group, future more RCTs are required to confirm the results. Standardization of the study features such as pain assessment tools is warranted for future RCTs in order to minimize the heterogeneity in studies. Future high-quality studies on novel methods of tooth isolation other than conventional latex rubber dam and clamp is also recommended.

## Conclusion

Within the limitations of the current study, no significant difference was found between the proposed methods for reducing pain and discomfort associated with rubber dam clamp placement in children and adolescents. A larger number of more homogenous studies regarding intervention methods and pain assessment tools needs to be conducted in order to draw stronger conclusions.

## Supplementary Information

**Additional file 1**.

## Data Availability

All data generated or analysed during this study are included in this published article [and its supplementary information files].
